# Pulse-by-pulse ultra-high resolution scintillation imaging of proton FLASH beams produced by a gantry-mounted synchrocyclotron

**DOI:** 10.1088/1361-6560/ae25b3

**Published:** 2025-12-11

**Authors:** S Murty Goddu, Scott Holloingsworth, Winter Green, Megha Goddu, Thomas R Mazur, Daniel Owen, Hailei Zhang, Yao Hao, Stephanie M Perkins, Arash Darafsheh

**Affiliations:** 1 Department of Radiation Oncology, WashU Medicine, St. Louis, MO 63110, United States of America; 2 Mevion Medical Systems, Littleton, MA 01460, United States of America

**Keywords:** FLASH, ultra-high dose rate, proton therapy, scintillation, synchrocyclotron

## Abstract

*Objective*. Radiation therapy at ultra-high dose rate (UHDR) has emerged in the past decade as a novel technique with potential to improve the therapeutic index through a phenomenon referred to as ‘FLASH effect’. Its reliable pre-clinical studies and safe clinical deployment would benefit from dosimeters with high spatiotemporal resolution, ideally with capability to perform pulse-by-pulse dosimetry for pulsed beams. Scintillation imaging has shown potential for high spatiotemporal resolution dosimetry that may be well-suited for UHDR beams. Our aim is to develop an ultra-high resolution scintillation imaging dosimetry system for characterization of pulsed UHDR proton beams on a pulse-by-pulse basis. *Approach.* A gantry-mounted proton therapy synchrocyclotron was used to deliver UHDR beams at ∼90 Gy s^−1^ average dose rate. A BC-408 plastic scintillator block (30 × 30 × 5 cm^3^), facing a high-speed camera inside an optically sealed housing, was used for range, relative dose-per-pulse, vertical spot position, Bragg peak (BP) widths, and beam size measurements. Another BC-408 plastic scintillator block (30 × 30 × 0.5 cm^3^), facing a mirror at 45°, was used to measure the spots’ lateral size and position. A high-speed complementary metal–oxide–semiconductor camera system, temporally gated with the accelerator’s pulse train, was used for imaging. Series of proton pulses with different ranges were delivered at various dose rates per pulse while the corresponding scintillation images were recorded to extract various beam parameters. *Results.* Individual image frames corresponding to each delivered pulse were successfully obtained at ∼0.08 mm per-pixel resolution. The integrated image intensity at the BP showed a linear correlation with the delivered charge-per-pulse. The scintillation signal agreed with the charge reading from the transmission ionization chamber within 1%. Our measurements did not demonstrate appreciable dose rate dependency of the ionization quenching in the scintillator. Range measurements on a pulse-by-pulse basis agreed within 1 mm of the programmed range and were confirmed with a multi-layer ionization chamber device. Spot sizes were measured within 0.3 mm of the expected values. *Significance.* Synchronized scintillation imaging can provide ultra-high spatiotemporal resolution dosimetry of pulsed UHDR proton beams desired for beam characterization. A strong correlation between measured scintillation intensity and beam current suggests that our system can be expanded for quantitative multidimensional dosimetry.

## 1. Introduction

Ultra-high dose rate (UHDR) radiation therapy (RT) at ≳40 Gy s^−1^ dose rate has attracted intense research attention in the past decade due to its potential to improve the therapeutic index compared to RT at conventional dose rates through a phenomenon referred to as ‘FLASH effect’, a differential effect observed between normal tissues and tumors (Esplen *et al* 2020, Vozenin *et al* 2022, Farr *et al* 2022b, Kim and Zou 2023, Di Martino *et al* 2024, Ghaznavi *et al* 2025). Successful clinical translation of FLASH RT requires stable UHDR radiation sources, reliable dosimeters, and understanding of its tissue sparing mechanism. The radiobiology mechanism responsible for FLASH effect is yet to be fully elucidated (Friedl *et al* 2022, Limoli and Vozenin 2023). To that end, multiple pre-clinical studies are ongoing. Investigating how various parameters including dose, average dose rate, and dose-per-pulse (for pulsed beams) influence FLASH effect requires high spatiotemporal resolution dosimeters due to the small fields involved and unique temporal aspects of the radiation fields (Romano *et al* 2022, Subiel and Romano 2023, Böhlen *et al* 2024).

To date, UHDR irradiation has been demonstrated with electrons (Schüler *et al* 2022, No *et al* 2023), photons (Rezaee *et al* 2021, Montay-Gruel *et al* 2022, Mansoury *et al* 2025), protons (Diffenderfer *et al* 2022, Kim *et al* 2022), and heavier ions (Weber *et al* 2022, Farr *et al* 2022a). Nevertheless, proton therapy is expected to be the main RT modality, in the foreseeable future, to treat deep seated targets at UHDRs (Verhaegen *et al* 2021, Ma *et al* 2025). Currently, human clinical trials are ongoing using proton FLASH RT (Mascia *et al* 2023).

The dosimeters commercially available for routine use in RT have been primarily designed, optimized, and characterized for conventional dose rate RT beams and lack the temporal resolution to measure individual pulses in pulsed beams (Romano *et al* 2022). Common ionization chambers and semiconductor dosimeters suffer from dose rate dependency which prompted development of dedicated ionization chambers (Zou *et al* 2021, Yang *et al* 2022, Zhou *et al* 2025) and diamond (Angelou *et al* 2024) detectors capable of operation in UHDR fields under certain conditions. Radiochromic films (Jaccard *et al* 2017, Patriarca *et al* 2018, Villoing *et al* 2022), thermoluminescent dosimeters (Montay-Gruel *et al* 2017), and optically stimulated luminescent dosimeters (Christensen *et al* 2021) have been used for FLASH RT dosimetry, but do not provide real time measurements (Darafsheh 2021). In this context, scintillation dosimetry is an attractive choice to provide two-dimensional (2D) dose measurement at high spatiotemporal resolution (Beddar and Beaulieu 2016, Darafsheh *et al* 2024). Scintillation dosimetry has found applications for *in vivo*, small field, and 2D dosimetry in RT (Beaulieu and Beddar 2016). Due to the fast response of scintillators, they are promising candidates for high temporal resolution dosimetry. High spatial resolution can be achieved through suitable optical systems or compact scintillators (e.g. optical fibers). Nevertheless, scintillation dosimetry is associated with its own challenges that stem from Cherenkov radiation and ionization quenching. The former is more significant in megavoltage photon and electron fields compared to proton beams (Darafsheh *et al* 2015, 2016). The latter, however, happens in radiation fields with high linear energy transfer (LET), such as proton beams (Wang *et al* 2012, Robertson *et al* 2013). Ionization quenching is manifested as under-response of the scintillator to the radiation dose within the Bragg peak (BP) due to non-radiative relaxation of excited states in the scintillator (Birks 1964).

Recently, we demonstrated feasibility of UHDR proton beam delivery for preclinical FLASH RT studies using a gantry-mounted synchrocyclotron in our clinic (Darafsheh and Bey 2025, Lowe *et al* 2025). Previously, we developed a scintillation imaging dosimetry system with high spatiotemporal resolution (0.16 mm-per-pixel) for dosimetric characterization of passive scattering (Goddu *et al* 2022) and pencil beam scanning (Goddu *et al* 2024) proton therapy beams at clinical dose rate. In this work, we aim to demonstrate the feasibility of scintillation imaging dosimetry for pulse-by-pulse characterization of UHDR proton beams, produced by a gantry-mounted synchrocyclotron, with ultra-high spatial resolution (0.08 mm-per-pixel). We demonstrate pulse-by-pulse correlation between the intensity of the images obtained by the camera with their corresponding charge-per-pulse obtained by a transmission ionization chamber. We also investigate ionization quenching in the scintillator as a function of dose rate.

## 2. Materials and methods

### 2.1. Proton therapy system

We used a pencil beam scanning gantry-mounted synchrocyclotron (S250i Hyperscan^TM^, Mevion Medical Systems, Littleton, MA, USA) in clinical operation to perform scintillation imaging experiments at conventional and UHDRs. UHDR beam delivery using the Hyperscan^TM^ system has been demonstrated (Darafsheh *et al* 2020, 2021). The Mevion FLASH product includes three FLASH-enabled components: (1) a FLASH-capable synchrocyclotron that enables the delivery of UHDRs, (2) a FLASH Research Kit that acts as a portable dosimetry system capable of measuring and controlling FLASH deliveries, and (3) a FLASH Research Mode in the treatment delivery system to ensure integrity of the clinical treatment delivery parameters. The FLASH Research Kit can be installed on a compatible system in ∼15 min and is completely removed when FLASH research is not being performed such that the clinical synchrocyclotron is unaffected. When in FLASH mode, the Mevion system can deliver ∼100 Gy s^−1^ average dose rate at the BP. Just like the clinical deliveries, the FLASH deliveries are pulsed with a nominal pulse frequency of 750 Hz, however, the pulse frequency can be modified between 600–780 Hz in FLASH Mode, and generally the duration of each pulse can be changed between ∼5–30 *µ*s to change the charge-per-pulse, and hence the average dose rate. The Mevion FLASH dosimetry system can terminate the beam based upon a set number of pulses or an accumulated charge in the FLASH-enabled transmission ionization chamber, which can be calibrated to the desired dose in a target with 2% accuracy at the calibrated dose rate and 5% linearity across the entire dose rate spectrum (Lowe *et al* 2025). UHDR deliveries are capable of being scanned across a 5 × 5 cm^2^ area with the FLASH kit.

Maximum energy of the protons prior to beam extraction is 227 MeV (32.2 cm range in water). For clinical use, proton range is modulated through a series of 18 polycarbonate range shifter plates within a motorized apparatus inside the nozzle. However, for FLASH RT delivery, a mechanical platform is mounted outside the nozzle and the beam’s range is changed by placing appropriate thicknesses of B_4_C and poly(methyl methacrylate) blocks with pre-determined water-equivalent thickness in the beam line (the dose rate remains within UHDR regime) (Darafsheh and Bey 2025).

### 2.2. Scintillation imaging setup

**Figure 1.**
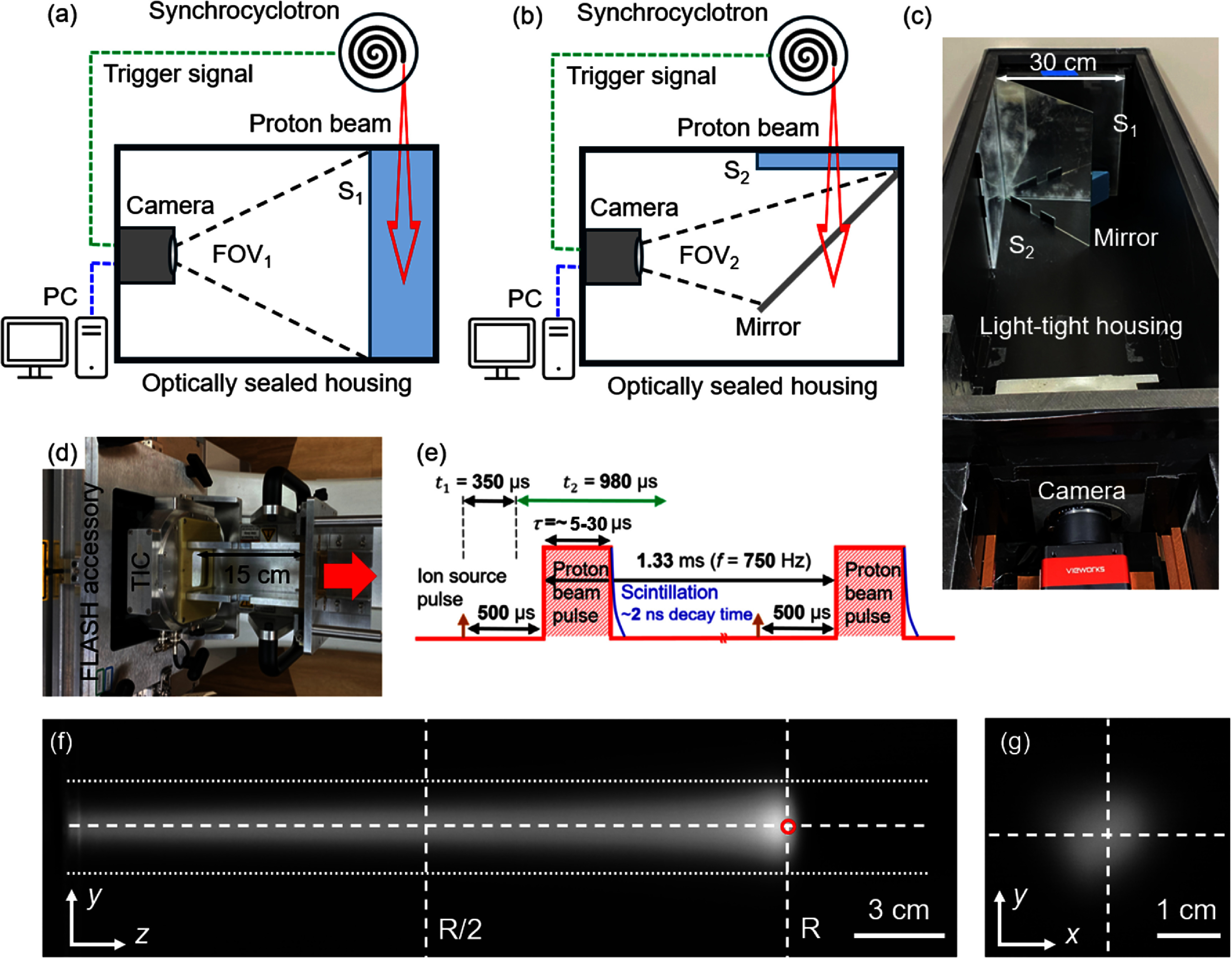
Schematic of the geometry of the synchronized scintillation imaging setup for UHDR irradiation measurements of (a) range (*S*_1_) and (b) spot size and position (*S*_2_). Photograph of the (c) imaging setup and (d) FLASH accessory mounted to the nozzle. Additional phantoms are placed in the 15 cm-long tray to change the proton range. The red arrow indicates the proton beam’s direction. Simultaneous presence of both *S*_1_ and *S*_2_ in (c) is for presentation only. During the measurements, either *S*_1_ is present, or *S*_2_ and the mirror as shown in the schematics. (e) Temporal radiation structure of the synchrocyclotron proton beam at ∼750 Hz. (f) A typical image obtained under *S*_1_ corresponding to a single spot with 25 cm range. (g) A typical scintillation image obtained through setup *S*_2_. TIC: transmission ionization chamber.

UHDR proton irradiation was done with the gantry at 90°. The geometry of our scintillation imaging setup is shown in figures 1(a)–(d). BC-408 (Saint-Gobain Crystals, Hiram, OH, USA) plastic scintillators (mass density *ρ* = 1.032 g cm^−3^, peak emission wavelength *λ*_peak_ ∼ 425 nm, rise time *t*_rise_ = 0.9 ns, and decay time *t*_decay_ = 2.1 ns) were used in this work. Scintillation imaging was performed in an optically isolated housing made of plywood with ∼3 mm water-equivalent thickness. To measure the proton range and relative intensity-per-pulse we used a 30 × 30 × 5 cm^3^ scintillator block (*S*_1_ in figure 1(a)). All faces of the scintillator block have been polished. For spot size and width measurements, a 30 × 30 × 0.5 cm^3^ scintillator block (*S*_2_ in figure 1(b)) was placed perpendicular to the beam direction, and a mirror placed at 45° reflected the scintillation images onto the camera’s sensor.

We used a high-speed complementary metal–oxide–semiconductor camera (VC-12MX2-M 330F, Vision Systems Technology, Vista, CA, USA) system with ‘Viewworks Imaging Solution 7’ capable of imaging at over 750 frames per second. The camera allows external pulse to trigger its shutter to open and close at programmed intervals. The camera was shielded against scattered neutrons by using dual-layer borated polyethylene blocks. The camera was located at ∼110 cm distance from the isocenter and perpendicular to the central axis of the beam. The isocenter is located at 205 cm distance from the exit of the cyclotron. The camera was placed on a thick copper block and cooled by frozen gel packs and miniaturized cooling fans in effort to reduce thermal noise.

The radiation output structure of our machine is shown in figure 1(e). Protons are delivered with macro pulses with 5–30 *μ*s temporal width separated apart by 1333 *μ*s (i.e. 756 Hz). The ion source pulse precedes each proton pulse by 500 *μ*s (i.e. it takes approximately 500 *μ*s for the injected ions at the center of the cyclotron to fully accelerate and exit the cyclotron). To perform pulse-by-pulse imaging, a gating module was used to synchronize the camera with the synchrocyclotron. The ion source pulse was used to provide a trigger for the camera’s shutter. The camera’s shutter opened 350 *µ*s after the ion source pulse is detected and remained open for 980 *μ*s ensuring imaging of a whole pulse in a single frame. This process repeated for all pulses. The images were saved in 8-bit tagged image file format. To create an average background image, images were acquired prior to and after each measurement, when the beam was off. The created background image was subtracted from the captured scintillation images prior to analysis. We used in-house scripts in MATLAB® (MathWorks, Natick, MA, USA) for image processing.

Geometrical scaling factors that convert pixel numbers to distance in vertical and horizontal directions were obtained through imaging a mm-scale grid pattern at the location of the scintillator block. In order to account for geometrical image distortions, we established non-linear scaling factors obtained by imaging a grid pattern.

Proton beams with water-equivalent ranges of 5, 10, 15, 18, 20, and 25 cm (corresponding to 79, 117, 147, 163, 173, and 196 MeV energy) were delivered to perform pulse-by-pulse evaluation of the range and relative dose-per-pulse using setup *S*_1_. Proton beams with charge-per-pulse of ∼10–35 pC were delivered to investigate the impact of average dose rate on the response of the scintillator. The corresponding dose rates at the BP are ∼30–100 Gy s^−1^. Figures 1(f) and (g) show typical images obtained from setups *S*_1_ and *S*_2_, respectively. After preprocessing, in-house scripts generated integrated depth intensity profiles by adding signal from all pixels in each column (within the two horizontal dotted lines in figure 1(f)) and assigning the integrated value to the respective depth. Then the peak depth was identified by the location of the maximum intensity. This process was conducted on a pulse-by-pulse basis. BP widths at 90% and 80% of the peak height were extracted for each pulse through the integrated depth intensity profiles. In addition, beam width at mid-range (*R*/2) and at the BP were computed by drawing vertical profiles and computing their full width at the half-maximum. The red circle in figure 1(f) (with ∼2.5 mm diameter (drawn not to scale)) shows a region-of-interest (ROI) over which the pixel values were integrated as a surrogate for intensity to correlate with the beam current. Depth dependent empirical correction factors were applied to correct for ionization quenching; the correction factors were obtained by dividing an integral depth dose (IDD) profile measured by an ionization chamber to that measured by the scintillation imaging system under the BP (Goddu *et al* 2022). We verified using radiochromic film dosimetry that the shape of the IDD did not change with the dose rate which allowed us to measure the IDD using a parallel-plate ionization chamber at a conventional dose rate (Darafsheh and Bey 2025). Since the Mevion system does not employ an energy selection system, the distal shape of the IDD profiles remain the same regardless of the proton energy (Goddu *et al* 2022, Darafsheh and Bey 2025). As such, we used the same correction factors for all ranges. We determined the distal 90% depth that represents the range of the beam.

UHDR proton beams with full energy and ∼30 pC-per-pulse were scanned over a 2 × 2 cm^2^ area with 1 cm spot spacing to measure the spot size and position on a pulse-by-pulse basis using scintillator *S*_2_ (figure 1(b)). After image preprocessing, in-house scripts determined the location of each spot. First, the grayscale image was converted to a binary image, and regions were labeled. Properties of these labeled regions, including the weighted centroid, were calculated, and the centroid of the largest, roughly circular region was identified as the spot. At this coordinate, horizontal and vertical profiles were drawn to determine the spot position and size (sigma) as shown in figure 1(g).

## 3. Results

### 3.1. Pulse-by-pulse relative dosimetry

**Figure 2.**
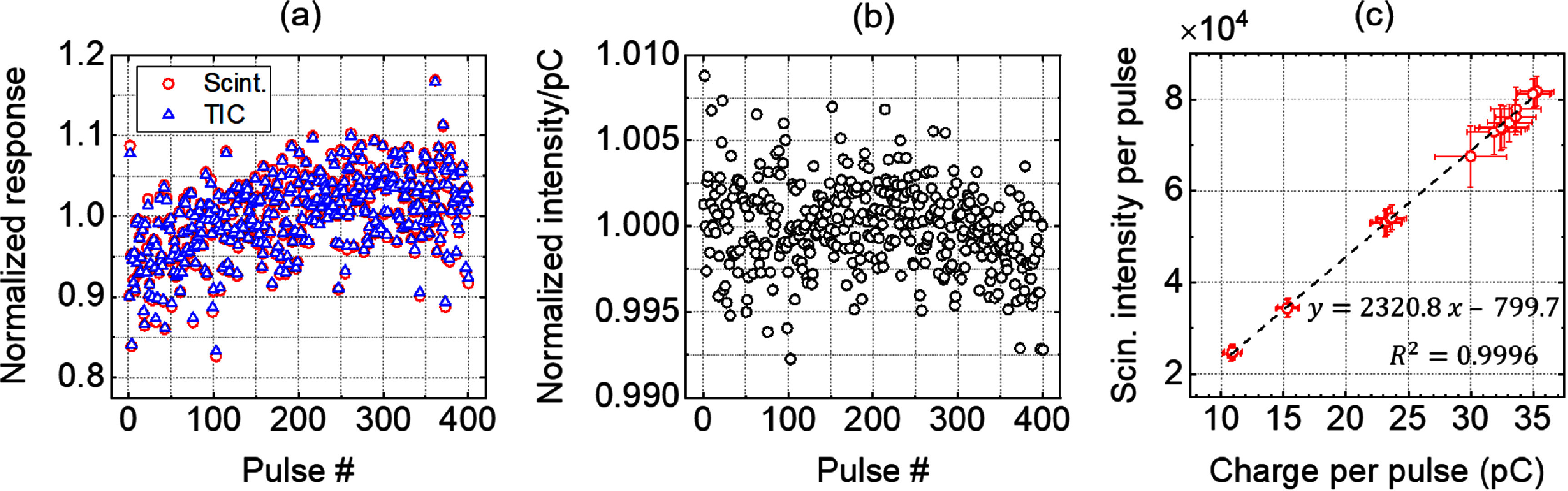
(a) An example of comparison between the integrated scintillation intensities of 400 images with their corresponding charge-per-pulse obtained from the machine log file measured by a transmission ionization chamber (TIC) demonstrating an excellent agreement. The values in panel (a) were normalized to their corresponding average value of the 400 pulses. (b) Demonstration of pulse-by-pulse relative dosimetry using the scintillation imaging technique in agreement with the machine log files. (c) Integrated scintillation intensity as a function of charge-per-pulse. Error bars show the pulse-by-pulse variations in pC/pulse in *x*-direction and scintillation intensities in *y*-direction. Each symbol in panels (c) represents a set of 400 pulses.

Since the camera was synchronized with the synchrocyclotron, each image corresponds to a delivered pulse. A total of 11980 pules in 30 datasets, collected under Setup 1, that include three different ranges were analyzed. The scintillation intensity captured in each 2D image, which is proportional to the dose-per-pulse, is encoded in the pixel values. We integrated the pixel values over an ROI centered at the BP location as a surrogate to the relative dose delivered by the synchrocyclotron. The ROI was a circle with 30 pixels in diameter (∼2.5 mm) along central axis and centered at the BP location as shown in figure 1(f). Integrated scintillation intensity of each frame was compared to the corresponding delivered charge-per-pulse recorded in the machine log files, as exemplified in figures 2(a) and (b) for 400 pulses. In figure 2(a), the integrated intensity and charge-per-pulse for each data point was normalized by their respective average value within the 400 pulses. Figure 2(a) demonstrates on average ±10% variation in charge-per-pulse across the 400 delivered pulses that is well-correlated with the transmission ion-chamber measurements. In some instances, the variation in dose-per-pulse reaches ∼15%. It can be seen in figure 2(b) that the majority of points are within 0.5% when the scintillation signal in each pulse was divided by the respective delivered charge, indicating an excellent correlation between the scintillation intensities and the charge-per-pulse. Figure 2(c) shows the integrated scintillation intensity as a function of charge-per-pulse (i.e. dose rate). The linear trend indicates the linearity of the scintillator signal with dose-per-pulse (instantaneous dose rate) from low dose rates up to the UHDRs studied in our work. Each circle in figure 2(c) represents the average image intensity in a data set containing ∼400 images (∼400 pulses).

### 3.2. Pulse-by-pulse beam characterization

**Figure 3.**
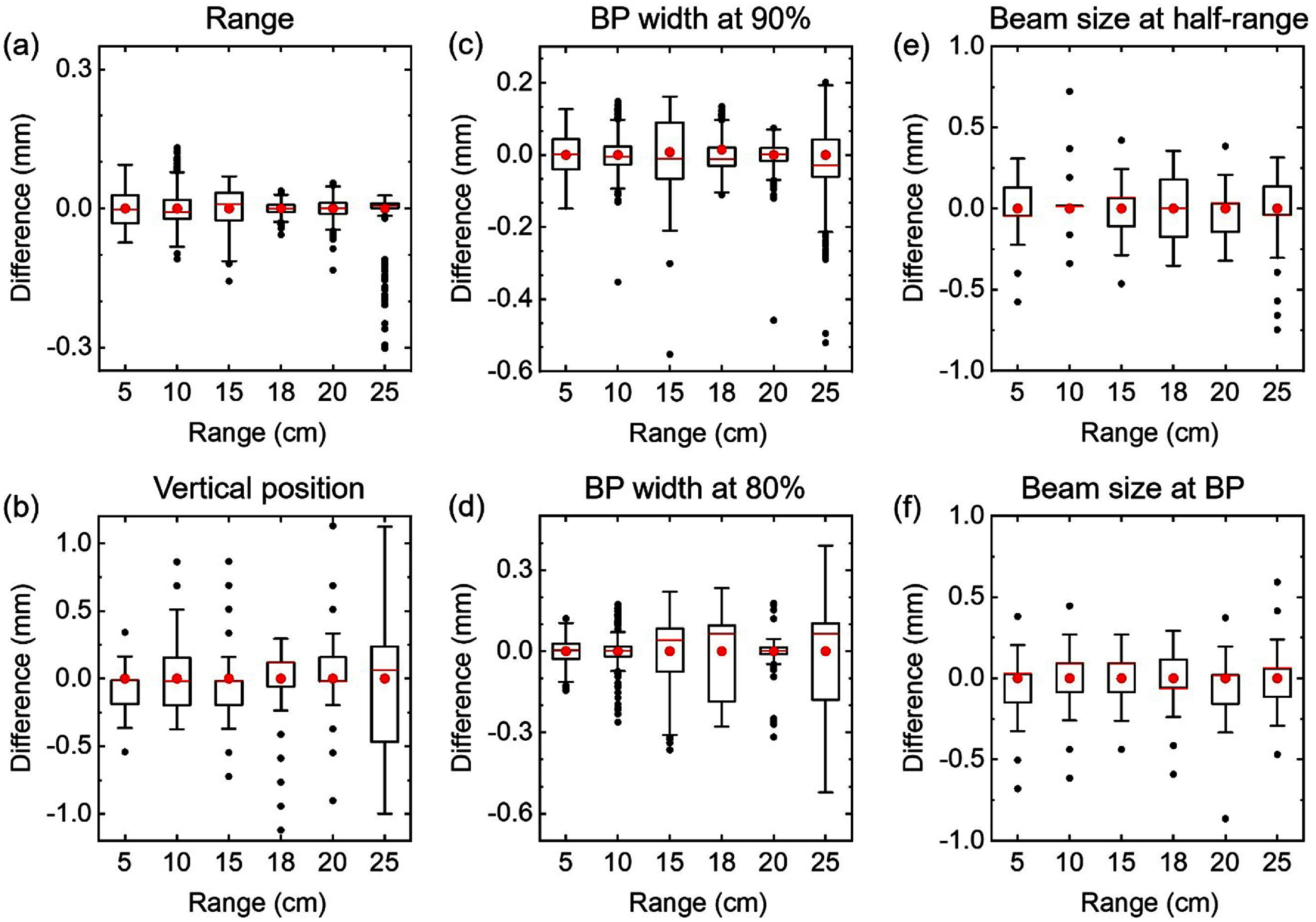
Pulse-by-pulse measurement of (a) range, (b) vertical position of the beam, (c) Bragg peak width at 90%, (d) Bragg peak width at 80%, (e) beam size at mid-range, and (f) beam size at the Bragg peak position. Boxes correspond to 25%–75% of the data, whiskers show range within 1.5 IQR, red lines are the median line, red circles are the mean, and black circles are the outliers. Due to the pixelated nature of the data, the distributions can be skewed in some cases.

A typical scintillation image obtained through the *S*_1_ setup is shown in figure 1(f). We extracted various beam parameters on a pulse-by-pulse basis through images obtained with scintillator *S*_1_. These parameters include proton range (distal *R*_90_), Bragg-peak width at 90% and 80% of the peak height, the beam’s width measured at mid-range and at the BP, as well as the BP’s vertical position. The variations presented in figure 3 are not absolute; they are computed from their respective average values in each set. The analysis of pulse-by-pulse range variation presented in figure 3(a) for three sets of proton beams each set with 6 different ranges with ∼300 pulses shows that the range variation is less than 0.1 mm in most cases. Figure 3(b) shows that the deviation in spot vertical position relative to the average value is mostly within ±0.5 mm. Figures 3(c) and (d) show the deviation with respect to the average value is mostly within 0.3 mm for the BP width at 90% and 80%, respectively. Figures 3(e) and (f) show the deviation with respect to the average value for the spots’ width is mostly within 0.5 mm at mid-range and BP, respectively.

**Figure 4.**
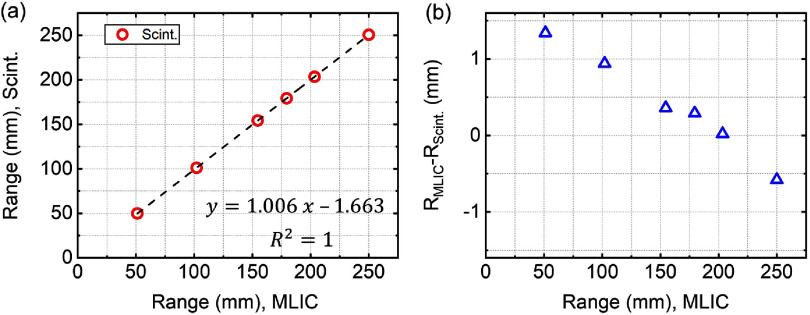
(a) Measured range through scintillation imaging (*R*_Scint._) against that using a multi-layer ionization chamber (MLIC) device (*R*_MLIC_) demonstrates a linear trend. (b) The difference between the two (*R*_MLIC_ − *R*_Scint._).

Figure 4(a) shows the average measured range for all delivered beams. A linear trend was observed between the measured range using the scintillator and the range measured by the multi-layer ionization chamber (MLIC) (Zebra, IBA). In most cases the measured range is within ±1 mm of the programmed range. Figure 4(b) shows that scintillator underestimates the range at shorter ranges (*R*_MLIC_ − *R*_Scint._ > 0) and overestimates the range at longer ranges (*R*_MLIC_ − *R*_Scint._ < 0). This systematic behavior requires further investigation.

### 3.3. Pulse-by-pulse measurement of proton spots’ position and size

**Figure 5.**
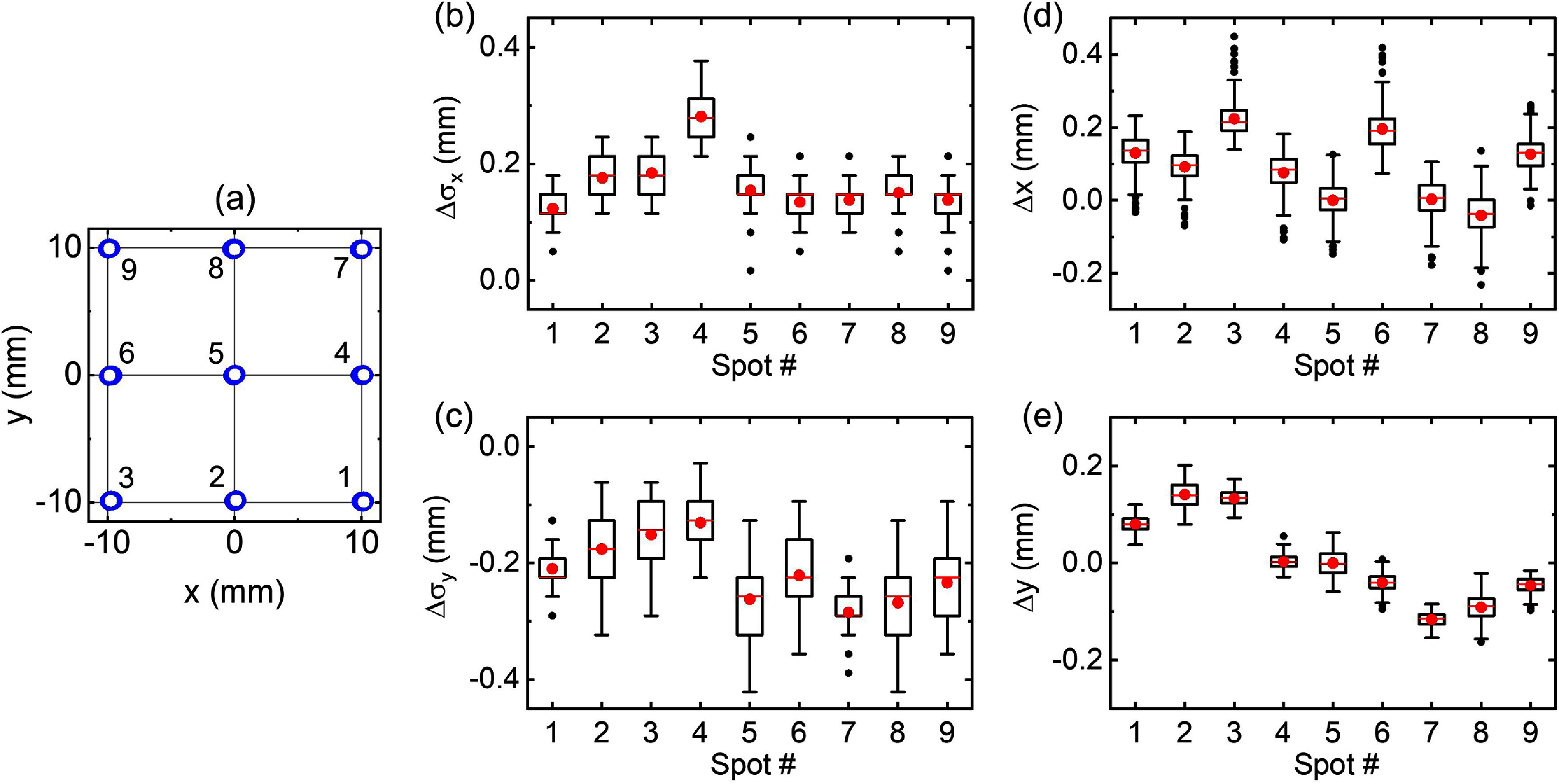
Pulse-by-pulse measurement of the spot size and position by the setup *S*_2_. (a) A composite map of 9 spots each with 150 pulses, separated apart by 10 mm, delivered across a 2 × 2 cm^2^ area. (b) and (c) Difference between the measured spot size (${\sigma _{{\text{Mes}}{\text{.}}}}$) and the expected size (${\sigma _{{\text{Exp}}{\text{.}}}}$) along the *x*- and *y*-axis for all pulses, respectively ($\Delta {\sigma _x} = {\sigma _{x{\text{,Mes}}{\text{.}}}} - {\sigma _{x,{\text{Exp}}.{ }}}$ and ($\Delta {\sigma _y} = {\sigma _{y,{\text{Mes}}.}} - {\sigma _{y,{\text{Exp}}.}}$). (d) and (e) Deviations from the expected position along the *x*- and *y*-axis, respectively. Boxes correspond to 25%–75% of the data, whiskers show range within 1.5 IQR, red lines are the median line, red circles are the mean, and black circles are the outliers. Due to the pixelated nature of the data, the distributions can be skewed in some cases.

Figure 5 shows pulse-by-pulse analysis of proton spot size and position measurement along the *x*- and *y*-direction using setup *S*_2_ for 1350 pulses delivered with full energy. Nine spots (each with 50 pulses), spatially separated by 10 mm distance, were delivered three times across a 2 × 2 cm^2^ area as shown in figure 5(a). A typical scintillation image of a single pulse of a single spot is shown in figure 1(g). The difference between the spot size (*σ*) measurement using our scintillation imaging system and the expected value measured using a commercial scintillation screen (Lynx, IBA) along the *x*- and *y*-axis for all 9 spots is shown in figures 5(b) and (c). The nominal corresponding values are 4.5 and 5.3 mm, respectively (Darafsheh and Bey 2025). The majority of pulses are within ±0.3 mm of the expected value. The systematic differences between the expected and measured values may be due to a slight misalignment of the mirror with respect to the camera.

The difference between the nominal and measured position along the *x*- and *y*-axis is shown in figures 5(d) and (e) with majority of the spots within ±0.2 mm of the corresponding nominal spot position.

### 3.4. Ionization quenching

**Figure 6.**
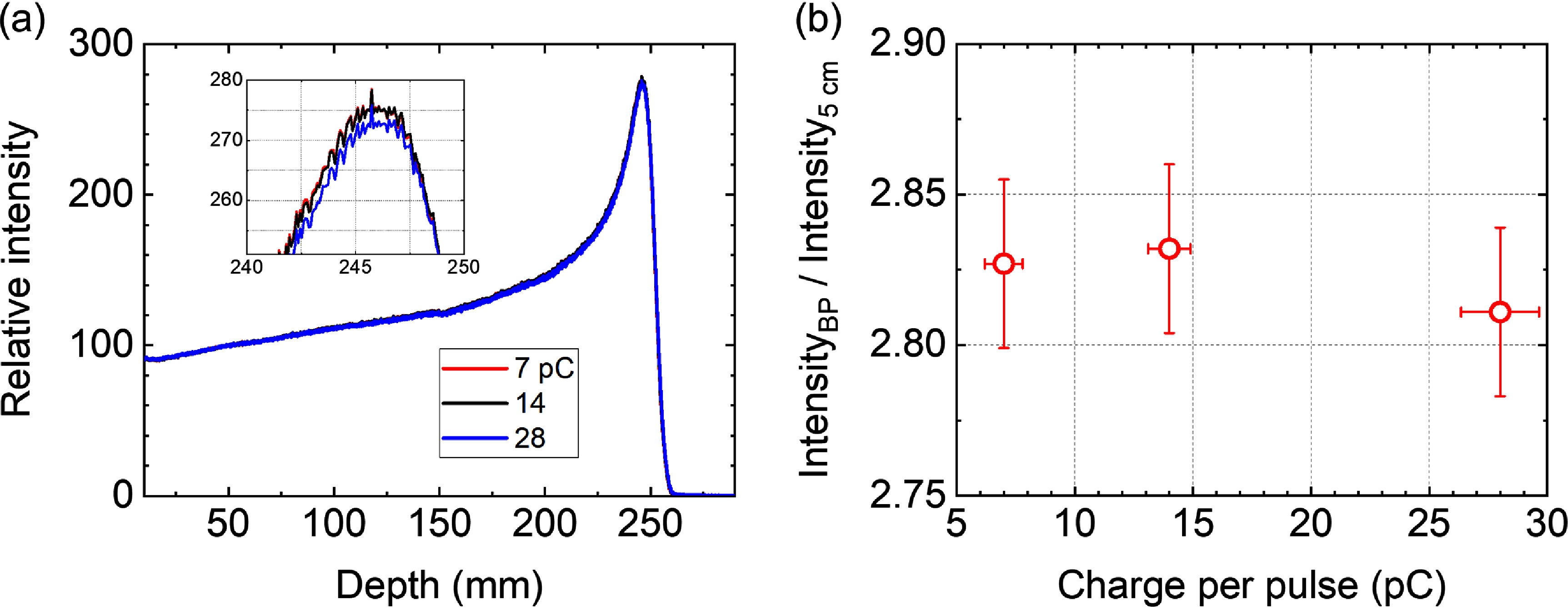
(a) Integrated depth intensity profiles obtained at different charge-per-pulses. The inset shows the expanded view near the Bragg peak. (b) Peak-to-plateau ratio of the scintillation intensity as function of charge-per-pulse.

Ionization quenching is manifested as an under-response of the scintillator around the BP, where the LET is high, in proton depth dose curves (Birks 1964, Wang *et al* 2012, Robertson *et al* 2013). To further investigate any dose-rate-dependency of ionization quenching of our scintillator at UHDRs, we compared integrated depth intensity profiles obtained at three average dose rates of ∼22, 43, and 86 Gy s^−1^ corresponding to 7, 14, and 28 pC-per-pulse in figure 6(a). In figure 6(b), we present the intensity of the images at the BP divided by that at 5 cm depth. These results as well as the linear trend in figure 2(c) do not point toward a noticeable dose rate dependency in ionization quenching, indicating that the same correction method can be applied in both low and UHDRs produced by our machine.

## 4. Discussion

Synchronized scintillation imaging of proton beams at UHDR dose rate using a high-speed camera allowed us to evaluate the FLASH RT beam delivery of a gantry-mounted synchrocyclotron on a pulse-by-pulse basis. An excellent agreement was observed between the brightness of each image (pixel value) and charge-per-pulse from the machine log file obtained through a transmission parallel-plate ionization chamber specifically designed for beam monitoring at UHDRs. Our results showed that the response of the scintillator was linear over a broad range of dose rates per pulse investigated in this work.

Setup *S*_1_, in which a thick scintillator is along the beam and perpendicular to the camera, allowed various beam parameter measurements at 750 fps corresponding to the 750 Hz pulse repetition rate of the synchrocyclotron. Analyzing each image frame allows pulse-by-pulse analysis of the radiation output of the machine. Pulse-by-pulse analysis indicated that the proton range, spot vertical position, BP width, and beam lateral size remained within 0.2 mm of the average value for majority of the case, indicating an excellent consistency of these parameters across a series of pulses. We noted that the relative dose per pulse varies ∼10%–15% in our proton therapy machine. Our scintillation imaging system was able to measure range within ±1 mm at 0.08 mm-per-pixel resolution. Since the intensity of each pulse (frame) was much greater under UHDRs compared to our previous work under clinical dose rates, it allowed us to capture the images without a need for binning, resulting in an increased spatial resolution by a factor of two in this work.

Setup *S*_2_, in which a thin scintillator is perpendicular to the beam’s axis and the light is reflected toward the camera through a 45°-mirror, allowed individual spot size and position measurement at 750 fps corresponding to 750 Hz pulse repetition rate of the synchrocyclotron. Pulse-by-pulse analysis of the images showed that the spots’ size along *x-* and *y*-direction remained within 0.5 mm of the corresponding expected value.

Scintillation imaging has been performed under UHDR proton beams using inorganic scintillators for surface dose measurements under an isochronous cyclotron (Clark *et al* 2024, Vasyltsiv *et al* 2025). In our work, the two arrangements using an organic scintillator with tissue-equivalent properties allowed beam characterization, under a synchrocyclotron, along the beam as well as perpendicular to the beam. Ionization quenching is a significant issue in scintillation dosimetry for high-LET radiation fields. Here, we did not observe a dose rate influence on quenching between UHDR and conventional dose rates allowing us to apply a dose-rate-independent correction factor to correct for ionization quenching. The quenching was approximately ∼20% across the dose rates studied in our work. In our proton therapy machine, since an energy selection slit is not employed to modulate the energy, the energy spectrum around the residual range remains similar for all energies, hence the slope of the distal shape of the BP remains the same (Goddu *et al* 2022, 2024). We exploited this fact to correct the depth dose curves for ionization quenching using an energy-independent depth-dependent correction factor.

Our 8-bit camera limited the signal-to-noise ratio (SNR) in which a difference of only 3 gray level values corresponded to >1% change. In principle, using cameras with higher dynamic range would enhance the SNR.

Here we used two scintillators to measure the 2D beam profile along the *xy-* and *yz*-plane. Using a multi-view scintillation imaging system, in principle, one can reconstruct the dose and dose rate in 3D which is the direction of our future work.

## 5. Conclusions

A scintillation imaging dosimetry system was developed and characterized for proton FLASH RT dosimetry. Our system demonstrated capability of measuring various beam parameters on a pulse-by-pulse basis at UHDRs. Our range measurement was within ±1 mm of the expected values. Comparing the scintillation intensities with the machine log files showed agreement within ±1%. It was found that ionization quenching did not depend on the dose rate under our beam. Our future work aims to optimize the system for use in 3D proton FLASH RT dosimetry.

## Data Availability

The data that support the findings of this study are available upon reasonable request.
